# The self-image of a mother in women with experience of miscarriage

**DOI:** 10.1192/j.eurpsy.2025.2397

**Published:** 2025-08-26

**Authors:** A. M. Bukinich, A. M. Konovalova

**Affiliations:** 1 Faculty of Psychology, Lomonosov Moscow State University; 2 Department of Pedagogy and Medical Psychology, Sechenov University, Moscow, Russian Federation

## Abstract

**Introduction:**

In the modern world, the problem of infertility is becoming more significant. In particular, the number of women with experience of miscarriage is increasing. Problems with pregnancy, frustration of the desire to become a mother influence the formation of a woman’s motherhood and self-image as a mother, which can lead to intrapersonal problems of a woman and to a distortion of care practices and child-parent relations in relation to the future child.

**Objectives:**

To study the features of the self-image as a mother in women with experience of miscarriage.

**Methods:**

A pilot study was conducted. Two methods were used in this study: “Unfinished sentences” (Sachs-Levy, mod. A.G. Dolgikh, 2018), method of semantic differential (spaces proposed by A.G. Dolgikh, 2018).

The study sample consists of 3 groups: 30 women with experience of miscarriage for medical reasons aged 25 to 35 years; 30 women aged 25 to 30 years who have no experience of pregnancy; 30 women raising children under the age of five.

**Results:**

The results of attitudes peculiarities psychodiagnostic study towards motherhood in women using the “Unfinished Sentences” technique demonstrate that generally more expressed positive attitude towards motherhood in general and attitude towards themselves as a future mother for the group of women with experience of miscarriage compared to other groups of women.

The self-image as a mother using the semantic differential method showed that the semantic universals of this image for both the entire sample and for a group of women with miscarriage experience are adjectives “reliability”, “caring”, “tenderness”.

The self-image as a mother semantic profiles comparative analysis revealed significant differences in the indicators of this image among the study groups. Women with the experience of miscarriage characterized themselves as more warm, more soft, more caring and more harmonious (p<0.05) compared with women from other groups. They also perceive the image of themselves as a future mother as lighter, more reliable, more anxious and more affectionate than women without experience of pregnancy and motherhood (p<0.05).

**Conclusions:**

Women experienced miscarriage are characterized by more idealized ideas about motherhood probably due to problems with pregnancy and frustration of the desire to become a mother.

**Disclosure of Interest:**

None Declared

